# New Tools for New Research in Psychiatry: A Scalable and Customizable Platform to Empower Data Driven Smartphone Research

**DOI:** 10.2196/mental.5165

**Published:** 2016-05-05

**Authors:** John Torous, Mathew V Kiang, Jeanette Lorme, Jukka-Pekka Onnela

**Affiliations:** ^1^ Brigham and Women's Hospital Department of Psychiatry Harvard Medical School Boston, MA United States; ^2^ Beth Israel Deaconess Medical Center Department of Psychiatry Harvard Medical School Boston, MA United States; ^3^ Department of Social and Behavioral Sciences Harvard TH Chan School of Public Health Harvard University Boston, MA United States; ^4^ Department of Biostatistics Harvard TH Chan School of Public Health Harvard University Boston, MA United States

**Keywords:** mental health, schizophrenia, evaluation, smartphone, informatics

## Abstract

**Background:**

A longstanding barrier to progress in psychiatry, both in clinical settings and research trials, has been the persistent difficulty of accurately and reliably quantifying disease phenotypes. Mobile phone technology combined with data science has the potential to offer medicine a wealth of additional information on disease phenotypes, but the large majority of existing smartphone apps are not intended for use as biomedical research platforms and, as such, do not generate research-quality data.

**Objective:**

Our aim is not the creation of yet another app per se but rather the establishment of a platform to collect research-quality smartphone raw sensor and usage pattern data. Our ultimate goal is to develop statistical, mathematical, and computational methodology to enable us and others to extract biomedical and clinical insights from smartphone data.

**Methods:**

We report on the development and early testing of Beiwe, a research platform featuring a study portal, smartphone app, database, and data modeling and analysis tools designed and developed specifically for transparent, customizable, and reproducible biomedical research use, in particular for the study of psychiatric and neurological disorders. We also outline a proposed study using the platform for patients with schizophrenia.

**Results:**

We demonstrate the passive data capabilities of the Beiwe platform and early results of its analytical capabilities.

**Conclusions:**

Smartphone sensors and phone usage patterns, when coupled with appropriate statistical learning tools, are able to capture various social and behavioral manifestations of illnesses, in naturalistic settings, as lived and experienced by patients. The ubiquity of smartphones makes this type of moment-by-moment quantification of disease phenotypes highly scalable and, when integrated within a transparent research platform, presents tremendous opportunities for research, discovery, and patient health.

## Introduction

The theoretical physicist Freeman Dyson, who was recently quoted by Dr Thomas Insel, the Director of the National Institute of Mental Health (NIMH) 2002-2015, has argued that “new directions in science are launched by new tools much more often than by new concepts” [1,2]. Smartphone and sensor technologies have recently emerged as novel tools in many fields of medicine, and there is growing literature in mental health on their potential to increase access to care [3], reduce stigma [4], improve diagnosis [5], and enable remote monitoring [6]. Despite the recent attention and interest in the potential of such data, less has been written about how to study, collect, store, analyze, and reproduce results of smartphone studies.

In this paper, we report on the development and early testing of Beiwe, a research platform designed and developed by the Onnela Lab at the Harvard TH Chan School of Public Health. The Beiwe platform is intended for biomedical research use, and it includes a study portal, smartphone app, database, and data analysis and modeling tools. Our aim is not the creation of yet another app per se but rather the establishment of a platform to collect research-quality smartphone raw sensor and usage pattern data. Our ultimate goal is to develop statistical, mathematical, and computational methodology to enable us and others to extract biomedical and clinical insights from smartphone data. In this paper, we focus on the app component of the platform and how it integrates across the other elements of the platform.

We also introduce the term “digital phenotyping” to refer to the “moment-by-moment quantification of the individual-level human phenotype in-situ using data from smartphones and other personal digital devices.” The data from these devices can be combined with electronic medical records and with molecular and neuroimaging data. In this sense, digital phenotyping can be viewed as a variant of deep phenotyping. Digital phenotyping is also closely aligned with the goals of precision medicine, which links new types of phenotypic data with genome data in order to identify potential connections between disease subtypes and their genetic variations [7]. Note that our definition of digital phenotyping is distinct from the “digital phenotype” that was introduced recently [8].

The data generated by increasingly sophisticated smartphone sensors and phone use patterns appear ideal for capturing various social and behavioral dimensions of psychiatric and neurological diseases. Given that the majority of the adult population in developed nations now owns and operates a smartphone, the act of measurement no longer needs to be confined to research laboratories but instead can be carried out in naturalistic settings in situ, leveraging the actual real-world experiences of patients.

While smartphones can be harnessed to offer medicine a wealth of data on disease phenotypes, the majority of existing smartphone apps are not intended for biomedical research use and, as such, do not generate research-quality data. While several commercial platforms collect similar data streams as Beiwe, they rarely allow investigators to access the raw data. Most offer only proprietary summaries of the data. This approach is problematic not only from the data analysis perspective, but it also makes it harder to replicate research. In a typical biomedical research setting, one first formulates the scientific question of interest, then determines what data are needed to address that question, and finally decides on a statistical approach needed to connect the collected data with the research question. This approach seems incompatible with platforms that do not allow access to raw data.

Finally, while many apps are able to collect data, without a research platform to support these data, results are difficult to analyze and reproduce. Because the Beiwe platform includes a flexible study portal, customizable app, scalable database, as well as an evolving suite of modeling and data analysis tools, researchers can use it for a diverse set of studies. Equally important, results can be re-analyzed and studies recreated and validated using the same data collection settings and the same data analysis tools as those in the original study, thus significantly enhancing the level of reproducibility and transparency in mobile health research.

In this paper, we document the development of the inaugural version of the Beiwe platform focusing on the app component, including implementation of its encryption, privacy, and security features. In addition to discussing features of the app, we also report on our ongoing testing and development of the platform to better understand its present capabilities and limitations. Digital phenotyping requires great concern for patient privacy and data security, and we discuss the medical risks and benefits for patients that we can foresee at this early state of the approach. We conclude with a protocol for a pilot study of the app in patients with schizophrenia and discuss how this line of research is able to inform the ongoing Research Domain Criteria Project of the NIMH [9].

### Background

The year 2014 marked a decisive moment in the history of mobile phones. For the first time, there were more active smartphone subscriptions globally than people on the planet [10]. Connected to this development, the rate of smartphone ownership has been steadily increasing over the past few years, and at present approximately 64% of the US adult population has a smartphone. Young adults between ages 18-29 now own smartphones at a rate of 85%, and ownership is expected to continue to increase across all populations with the majority of new phones sales being smartphones instead of technologically less advanced feature phones [11]. Smartphone ownership has also been investigated among psychiatric patients, and early findings based on a relatively small sample size suggest that psychiatric patients are no less likely to own a smartphone than the average person and are not opposed to using their own phones to monitor their mental health [3].

The final aspect that makes it feasible to collect sensor and usage data is that smartphones can be programmed using third-party apps. Thousands of mental health apps have become available to consumers in the past few years, and they have usually been positioned within the quantified-self or mHealth movements. These apps have various functionalities, but very few of them are intended for research use. We can draw an analogy here between digital phenotyping and DNA sequencing based on the intended use of these technologies. Next-generation DNA sequencing methods have made it inexpensive for anyone to learn about their ancestry, but research-quality sequencing typically requires much greater sequencing depth and remains more costly to perform. Like genetic research tools that sequence each nucleotide in a gene, chromosome, or an entire genome, smartphone apps intended for research use need to record and store all relevant data. They also need to be customizable to the needs of any given study by allowing the researchers to specify what data are collected and how they are collected, such as the sampling frequency of the accelerometer. Finally, they need to incorporate data analysis and modeling as clinical insights come from combining data with analytical methods. The goal of many of the existing mHealth apps is to collect and report summaries of either user-reported data or data collected from some of the phone’s sensors.

Both psychology and psychiatry have a history of quantifying human behavior in situ. Briefly, ecological momentary assessment (EMA) refers to a collection of methods used in behavioral medicine research for participants to report on symptoms and behaviors close in time to experience and in the participant’s natural environment [12]. It is related to the experience sampling method (ESM) [13]. Both EMA and ESM rely on self-reported accounts of behavior, and both have traditionally required the use of specialized tools from notebooks to electronic devices for the duration of the study. For example, some studies used personal digital assistants, but since this technology never really took off, subsequent studies have not been able to exploit it. While laudable in their inventiveness, traditional EMA/ESM approaches do not lend themselves to long-term follow-up due to the burden to the patient of carrying an additional device that may not have any direct functionality. They also scale poorly to large cohorts because that would require access to a large collection of study devices. Because both psychiatric and neurological disorders can be long-term illnesses and show significant variation in their symptomatology from person to person, there are clear benefits to being able to monitor a large group of patients over extended periods of time without any additional instrumentation. Some recent implementations of EMA/ESM make use of smartphones, but so far, it is with the apparent goal to replicate more traditional forms of EMA/ESM in a more convenient way.

Given these developments, how is smartphone-based digital phenotyping distinct from EMA/ESM? The short answer is that the purpose of digital phenotyping is not to implement surveys (although surveys can be incorporated into it) but to collect and analyze large quantities of various types of social and behavioral data that capture the subjects’ lived experiences and their interactions with people and places. Among others, digital phenotyping encompasses the collection of spatial trajectories (via global positioning system [GPS]), physical mobility patterns (via accelerometer), and audio samples (via microphone).

## Beiwe Research Platform

### Overview

**Figure 1:**
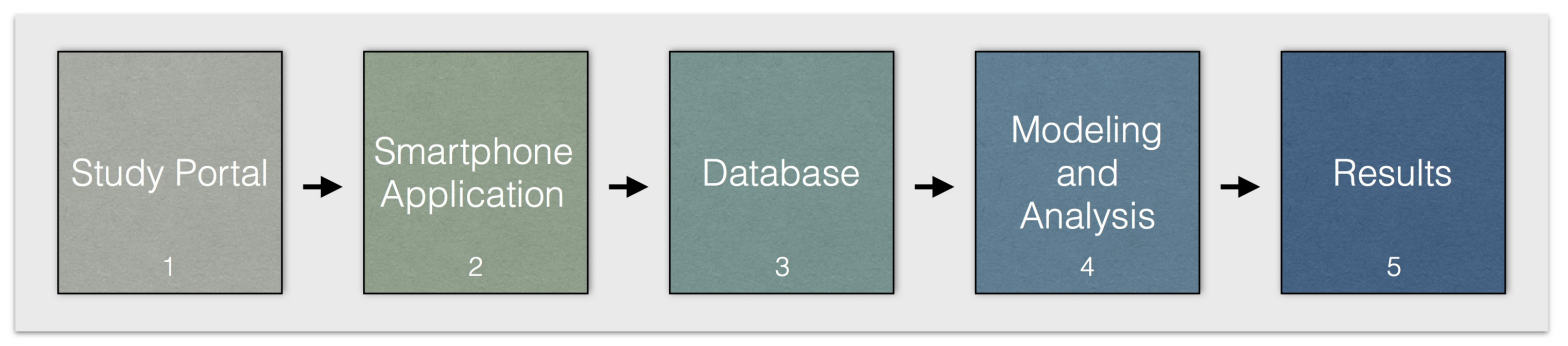
Workflow on the Beiwe platform.

The Beiwe platform was designed with the philosophy that every clinical or biomedical study starts with specific research questions and has its distinct data collection and analysis requirements. The workflow on the platform consists of several steps (see Figure 1) and reflects the natural flow of a research project. Our current set-up makes it possible to customize the app for a given study using a Web-based study portal. Among other things, this allows the investigators to set up any number of surveys, specify the content of the app, specify which sensors are used for data collection, and how they are sampled. This results in the generation of a new study specific app. Collected data are immediately encrypted and buffered on the device until a Wi-Fi connection becomes available, at which point the data are uploaded to a database on the study server. The data can be analyzed using an evolving suite of software, to be released at a future date to the public domain, which combines more traditional biostatistical analyses with machine learning techniques capable of dealing with millions of observations.

**Figure 2:**
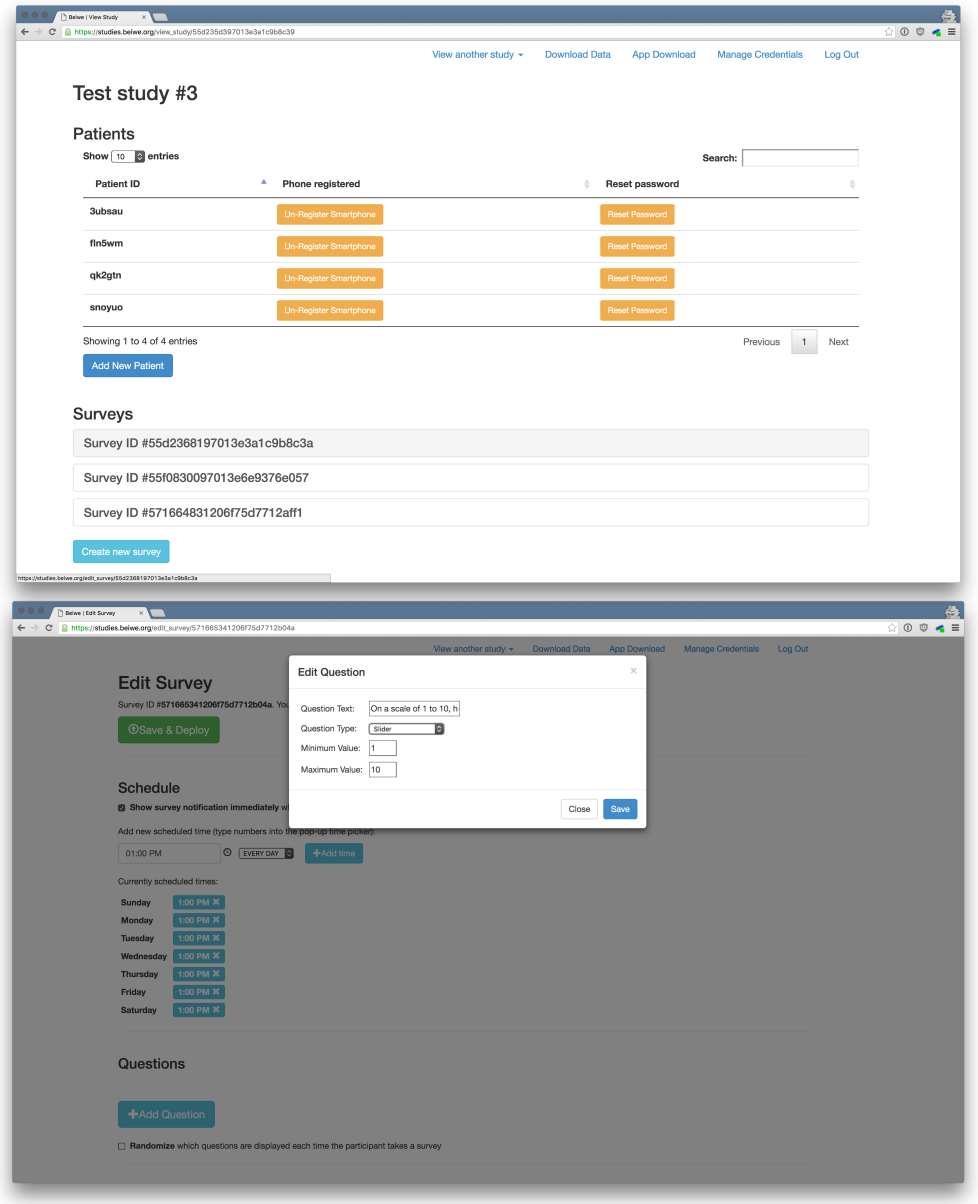
The Beiwe research administrator panel allows researchers to add new patients to a study (top) and create surveys and customize survey deployment (bottom).

The online study portal (see Figure 2) is used to create new studies and to manage existing ones. As part of the process of creating a new study, investigators customize all aspects of phone data collection, including what sensor data are collected and how frequently they are sampled. This is also where investigators can program any number of smartphone-based surveys, specify their timing and content, and enter any informational text that appears on the app screen (eg, text used to solicit audio samples and text describing study objectives). As a consequence, each study has its own version of the app with its own unique features.

Once a study has been created, investigators can generate unique participant IDs for subjects and perform basic administrative operations, such as remove a subject or reset a password. The only identifier linking subjects to their data in the platform is the participant ID. When a subject downloads the app, the platform uses the participant ID to determine which virtual version of the app to download and install. Beiwe uses a store-and-forward architecture for managing data, meaning that data are buffered on the device until Wi-Fi becomes available, at which point the data are uploaded to the server database and expunged from the device. Direct identifiers in the collected data are hashed, and data are always encrypted, as discussed in more detail below.

The Beiwe platform is currently available to only a small group of researchers. However, to stimulate further research into digital phenotyping and to promote transparency and replicability of that research, we envision releasing both Beiwe and a software suite for analyzing data collected by Beiwe to the public domain at a future date. Finally, for those curious, Beiwe is the name of the solar deity of the Sami people, an indigenous Finno-Ugric people. The name references the complex yet powerful relationship between personal experiences, physical environment, and mental health—all different aspects of the lived experiences that the Beiwe platform seeks to bridge.

### Surveys

Surveys have a long history in the social sciences, psychology, and medicine, in particular psychiatry. Paper has been the traditional medium of delivering surveys, and only more recently have Web-based online surveys become more common [14]. Smartphone-based surveys, like Web-based surveys, make it possible to record the exact start and end time of surveys, but smartphones can also capture the physical location of the subject via GPS at the time the survey was taken [15]. Given that human behavior is highly context sensitive [16], being able to localize the surveys in both time and space would seem to generate new research opportunities. Previous attempts to have patients conduct EMA using pencil-and-paper methods have noted high rates of backfilling and unreliable data [17]. Current research has instead underscored not only the feasibility, but also utility of having patients, including those with schizophrenia, complete surveys on smartphone platforms [18,19].

Beiwe implements several different types of surveys that can be customized using its Web panel. The responses to survey questions can take any of the following forms: (1) checkbox: multiple options can be selected from a list, (2) radio buttons: one option is selected from a list, (3) numerical slider: a numerical slider bar can be moved along a line, and (4) free response: text can be entered directly into the survey field.

Currently Beiwe implements any number of surveys on any schedule, which allows for maximal flexibility. For example, one could implement a short daily survey and a more detailed weekly survey. Whenever a new survey is available, the app notifies the subject by bringing up a survey prompt, and Beiwe also quantifies the amount of time that passes between the survey notification and the time the survey is actually taken. While Beiwe does not give any feedback to subjects based on their passive data streams, it does display a simple graph of the subject’s responses to numerically quantifiable questions from the previous week.

### Characteristics of Research Platform and Research Data

The characteristics of scientific research platforms like Beiwe are quite distinct, on multiple levels, from the existing commercial mHealth apps. The research app component of Beiwe functions primarily as a data collection engine, meaning that it attempts to collect the specified data streams using the specified sampling scheme as accurately as possible. Second, the data analysis is not done in real time on the phone but instead takes place on a dedicated server, which enables the use of sophisticated data analysis techniques that are not possible to perform on the phone itself. Third, Beiwe stores data on the phone only temporarily, and whenever a Wi-Fi connection is established, it uploads the data to the server and expunges the data from the device. Fourth, while many commercial and research applications attempt to give subjects feedback, Beiwe attempts to construct social and behavioral phenotypes with minimal user interference and is not at present intended for behavioral interventions. In order to minimize the impact of measurement on what is measured, Beiwe gives only very minimal feedback to the subject in order to avoid behavior change that could result from this feedback.

Arguably the most important aspect of a research platform is the collection of raw sensor and phone use data. Reliance on data summaries, especially on proprietary data summaries, is problematic for two reasons related to data analysis and replicability of research. First, smartphone data are high dimensional, longitudinal, exhibit interstream and temporal correlations, and are typically sampled at adaptive rates depending on the state of the phone (active vs sleep). This has the implication that one needs to exercise extreme care when considering different data summaries and different data analytic strategies. Proprietary data summaries rely on undisclosed assumptions, and they are fixed before either the scientific questions or the statistical approach have been formulated. In the best case, this compromises the validity of the statistical analyses, and in the worst case, it leads to research that is driven by what data summaries happen to be available rather than research driven by authentic research questions. Second, aside from analytical challenges, collection of raw data means that results can be re-analyzed retroactively and studies can be replicated and validated using the same data collection settings and the same data analysis tools as those in the original study. This aspect significantly enhances the level of reproducibility and transparency in research carried out using mobile devices. The fact that proprietary data summaries can be changed at whim without disclosure means that even using the same summary from the same vendor is no guarantee that the metric is the same.

We divide all data collected by Beiwe into two categories: active data and passive data. We define active data as data that require active participation from the subject for its generation, such as surveys and audio samples (more below). In contrast, we define passive data as data that are generated without any direct involvement from the subject, such as GPS traces and phone call logs (more below). We also use the term “data stream” to jointly refer to all the different types of continuously sampled smartphone passive data.

We note that there are at least three factors in any given study that might influence the decision regarding what type of data to collect and how to collect it. First, the decision regarding what types of data to collect and what specific parameter values are optimal for each type of data should be driven by the scientific questions at hand. Second, in order to protect patients’ right to privacy, it is pertinent to collect only the type of data that can be brought to bear on the specific scientific questions that are being investigated. Third, collection of active and passive data increases the phone’s consumption of electricity. If the phone sensors are sampled too frequently, the app can drain the phone battery in a short time.

### Data Encryption, Security, and Privacy

Beiwe was designed to collect large quantities of social and behavioral data and as such, both security and privacy are high priorities. Yet privacy in the digital world can be complex as much data may actually be outside the legal protection of the Health Insurance Portability and Accountability Act (HIPAA) [20], and clinicians may have a difficult time keeping up to date on security and privacy policies. For these reasons, Beiwe was designed on the premise that identifying data, such as phone numbers, should be protected through hashing and that, in addition, all data should be encrypted at all times. Beiwe uses a store-and-forward architecture for managing data, meaning that data are buffered on the device and as soon as a Wi-Fi connection is available, all data are securely transmitted in a HIPAA-compliant manner (details below). Using Wi-Fi for data transfer has the obvious benefit that it does not use up a subject’s 3G/4G data plan, which is important given the large quantities of data collected. Delays in data transfer, until a subject is connected to Wi-Fi, will not alter data collection or quality.

During study registration, the platform provides the smartphone with the public half of a 2048-bit RSA (Rivest-Shamir-Adleman) encryption key. With this key the device can encrypt data, but only the server, which has the private key, can decrypt it. Thus, the Beiwe app cannot read its own data, so even if a phone is lost or stolen, no information is compromised. The RSA key is then used to encrypt a symmetric Advanced Encryption Standard key for bulk encryption. These keys are generated as needed by the app; therefore, they are not stored anywhere and must be decrypted by the study server before any data can be recovered. Our current set-up does not use local servers but instead relies on cloud computing from Amazon Web Services, where at present we use an Amazon EC2 instance as the study server and an Amazon S3 instance for data storage. In our current configuration, data received by the EC2 server are re-encrypted with a master key provided for the given study and then stored on an Amazon S3 instance, an industry-standard secure storage platform housed in guarded data centers.

The main identifying information collected by Beiwe is phone numbers and the unique media access control (MAC) addresses of Wi-Fi and Bluetooth devices. The app uses the industry standard SHA-256 algorithm to carry out a one-way hash from a phone number or MAC address to a surrogate key. This mapping is always the same, meaning that a given phone number or MAC address will always be replaced by the same surrogate key. Importantly, it is impossible to undo a hash (in mathematical terms, the hash function is not invertible), which ensures that identifying information remains secure.

Finally, unrelated to data itself, but closely related to patient safety more generally, the app includes an easy-to-use, in-case-of-emergency feature that enables the patient to request immediate help from a medical professional. These emergency numbers and the numbers of clinical research coordinators are customizable for each study and are entered into the Beiwe app during a subject’s registration in a study.

### Features of Beiwe App Data

The following provides a description of some of the active and passive data types collected by Beiwe 1.0 running on a Samsung Galaxy S4 running Android 5.0. Of note, a version of the Beiwe app that will run on Apple’s iPhone is currently under development and will be completed early 2016. Given the differences across the operating systems, the data streams collected on these two platforms will not be identical, but every effort will be made to standardize them. One notable difference is that, at the time of writing, Apple’s iOS does not allow the extraction of phone call and text message logs, so the data streams collected from Android platforms will be somewhat richer. Apart from these differences, both the Android and iOS versions use the same Web study portal, database, and modeling and data analysis tools.

#### Global Positioning System and Accelerometer

**Figure 3:**
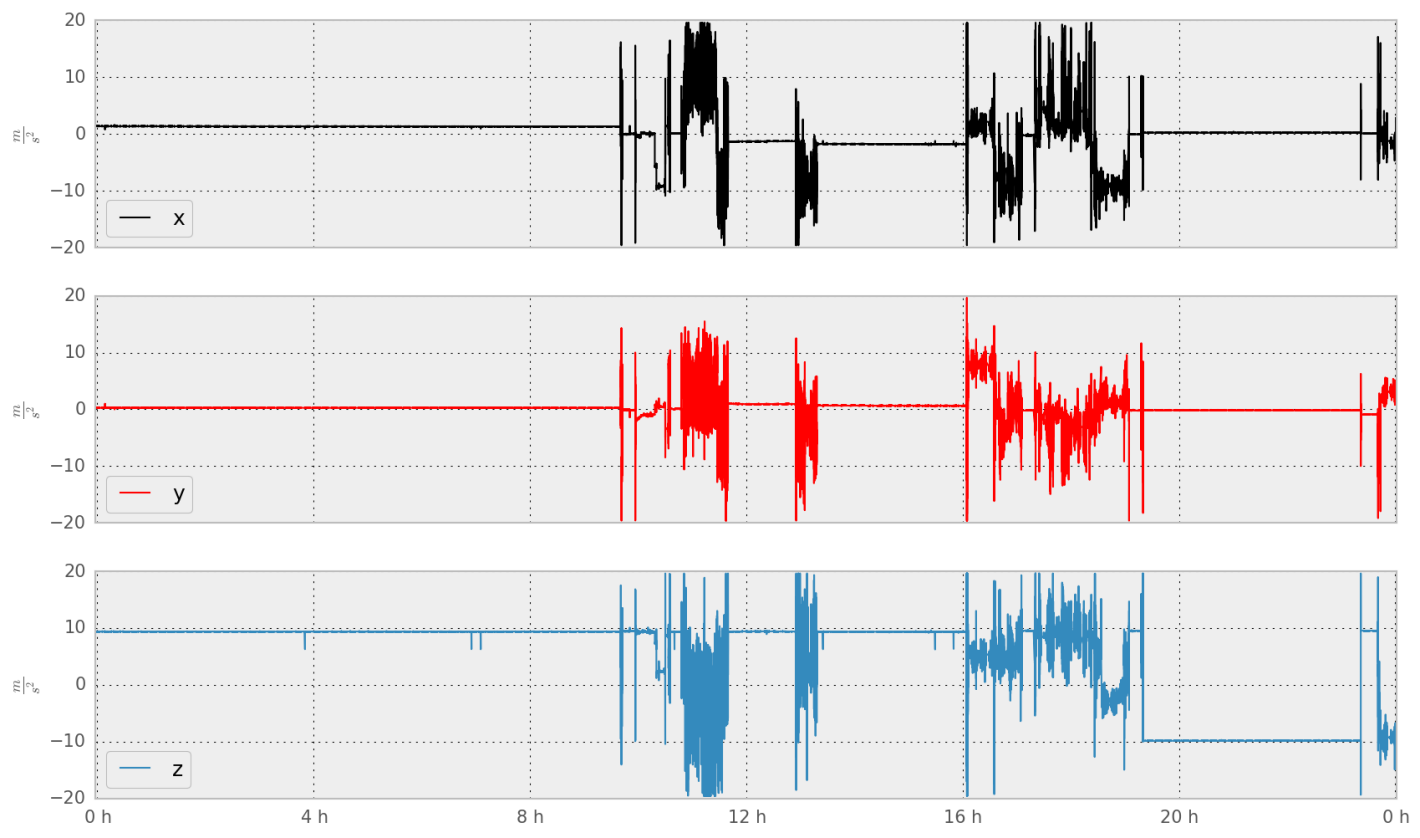
Sample accelerometer data collected by Beiwe (24 hours).

**Figure 4:**
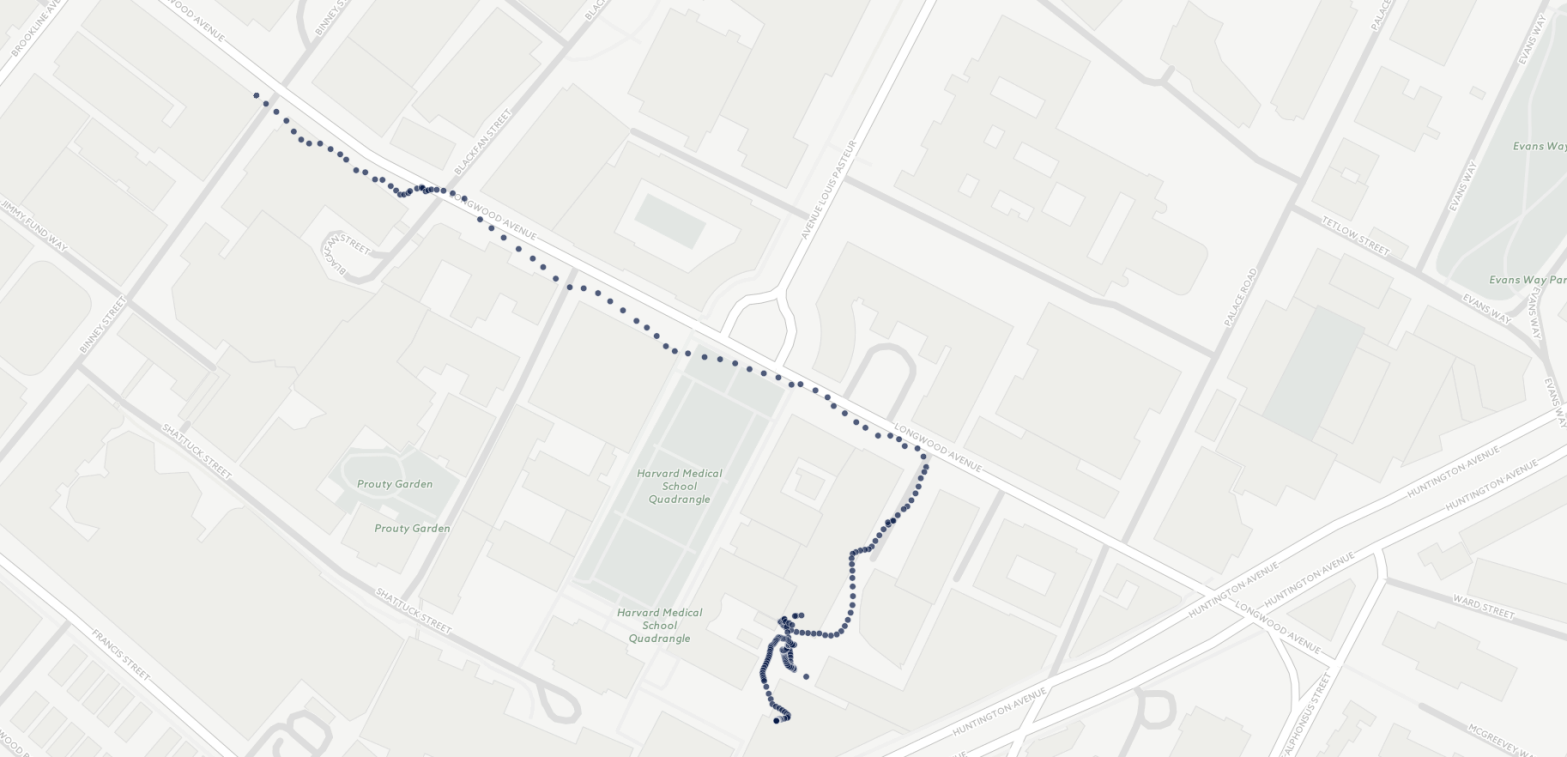
Sample GPS data collected by Beiwe over a 5-minute interval.

Beiwe uses the GPS chip to record the spatial location of the phone over time. GPS sampling is typically periodic, occurring at pre-specified regular intervals, but the operating system may not always allow for the GPS query to be implemented in order to prevent battery drainage (eg, if the phone is inferred to be stationary). Beiwe also records the phone’s accelerometer data to produce a history of the phone’s movement, specifically a trajectory of its acceleration, which can be used to quantify movement and mobility patterns. For both GPS and accelerometer data (see Figures 3 and 4), the optimal sampling rates will depend on the specific scientific questions, as well as how much battery drainage is acceptable in any given study, which is why the sampling schedule for both sensors is fully customizable. More specifically, the durations of the time intervals when each sensor is on and off can be specified precisely. When the phone is in motion, the operating system samples GPS at approximately one data point every second, or at 1 Hz. GPS longitude and latitude data are most often accurate to within 30-50 feet. While battery drainage is always a concern with GPS as well as accelerometer, in our internal testing, the battery life still lasted an entire day for various sampling settings. The accelerometer chip in most smartphones has a sampling rate between 5-100 Hz, depending on the device model and the mode the device is in (eg, screen on or off). The accelerometer records acceleration along the x, y, and z axes and reports acceleration in units of meters per second squared (m/s^2^).

Using schizophrenia as an example, it is possible to envision GPS and accelerometer data having significant clinical potential. Recent studies have collected GPS data from patients with alcohol use disorder to better support patients [21], and others have underscored the correlation of GPS data with depressive symptoms [6]. It is possible that certain states of schizophrenia, such as paranoia, may be correlated with decreased or altered patterns of movement. Of note, these two studies utilizing GPS data did not report significant battery issues related to GPS use. Accelerometer data could be useful in helping monitor for neurological side effects of medications, such as tremors present when the subject is holding the phone to make a call and also in understanding activity states (eg, running vs walking) [22].

A visualization of a spatial trajectory is available, constructed from GPS samples over a 5-minute interval, collected by Beiwe [23] (figure and animation were created with software from CartoDB).

#### Phone Call and Text Message Logs

Mobile phone data, in the format of anonymized call detail records obtained directly from phone operators, have now been used for about a decade to study social and communication networks, communication dynamics, spatial mobility, and more [24-29]. Although collecting phone call and text message logs via a smartphone app would at first seem to add little to this burgeoning literature, the fact that they can be combined with other types of sensor data makes them increasingly valuable from a research point of view.

**Figure 5:**
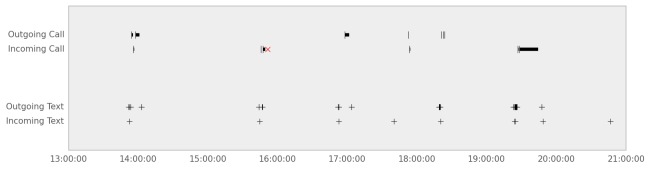
Sample data showing a record of incoming and outgoing text messages and phone calls recorded by Beiwe (duration of phone calls is noted by length of the corresponding line, and text messages are noted by the + symbol).

The Android version of the Beiwe app collects phone call and text message (SMS) logs detailing communication events between subjects and their social contacts (see Figure 5). The phone numbers are one-way hashed to render them non-identifiable (see Data Encryption, Security, and Privacy for more details). The communication logs contain only communication metadata, that is, data pertaining to the communication events themselves, but no actual content of communication, either auditory or textual, is stored by the app. The text message log includes the date, time, indicator for whether the text was sent or received by the subject, hashed phone number of the other party, and message length quantified in number of characters. The phone call log includes the date, time, hashed phone number, call type (incoming, outgoing, missed), and duration in seconds for each call.

Considering schizophrenia, it is possible to envision that information from these anonymized call and text messages logs may be a potential marker for social activity. Negative symptoms in schizophrenia can often blunt social activity and the Beiwe platform may offer early detection of worsening symptoms as well as response to treatments targeting negative symptoms.

#### Wi-Fi and Bluetooth

**Figure 6:**
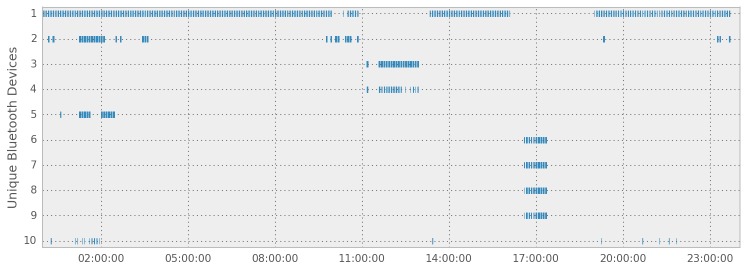
Sample Bluetooth data collected by Beiwe demonstrate its ability to detect and log nearby signals over the course of a day.

**Figure 7:**
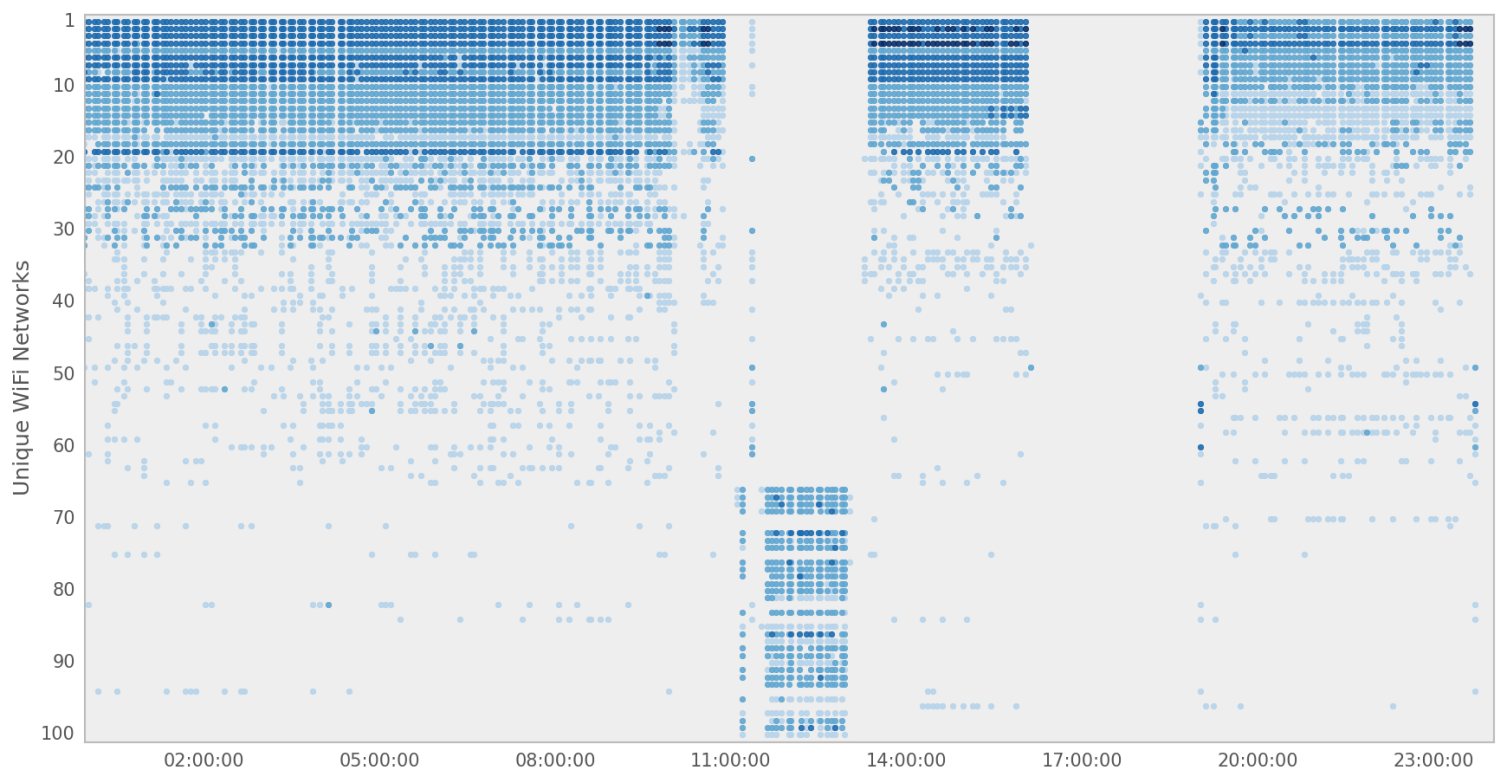
Beiwe scans for nearby Wi-Fi signals throughout the day and records their hashed MAC addresses and signal strengths.

The Beiwe app scans both Wi-Fi and Bluetooth signals that are used to improve estimation of its spatial location, especially while indoors, and to determine whether the subject is nearby other people (see Figures 6 and 7). The Wi-Fi scans cover both the 2.4 GHz and the 5 GHz frequency bands, whereas Bluetooth operates on the standard 2.4 to 2.485 GHz frequency band. Each Wi-Fi and Bluetooth device has its unique identifier, the MAC address, which Beiwe hashes using the approach described above (see Data Encryption, Security, and Privacy). Signal strength data (in dBm) are collected for both data streams, which can be used to estimate the physical proximity of the subject to the transmitting device. The rate of both Bluetooth and Wi-Fi scanning can be adjusted depending on the research goals of the study, but as default settings, we sample Bluetooth for 1 minute every 5 minutes and Wi-Fi networks are recorded (instantaneously) every 5 minutes. A future version of Beiwe will have the capability to use Bluetooth to incorporate additional study instrumentation, such as wearable wristbands, to complement the smartphone-based measurements. However, the current version uses Bluetooth solely to learn about the proximity of other Bluetooth devices, such as smartphones.

Taking schizophrenia as a use case, Bluetooth and Wi-Fi data offer several potential uses. One can envision instrumenting an entire family with a schizophrenic family member with the Beiwe app. Smartphone Bluetooth transmitters could provide new levels of detail on how an individual with schizophrenia spends time with various family members. In addition, Bluetooth beacons—small devices that send out frequent pings using the Bluetooth frequency band—could be placed in different rooms of the home to give a new level of detail on how these individuals use their personal space and whether changes in space use may be correlated with illness. Similarly, Wi-Fi can provide precise information on specific venues that the subject visits, such as bars, and this information, with the patient’s consent, could be used to investigate whether time spent at these venues is correlated with symptoms.

#### Audio

Beiwe can also record audio samples from subjects (see 8). The investigator specifies the maximum duration of the recording, at which point the recording terminates, but the subject can also stop the recording at any time before reaching the end. After completing the recording, the subject has the option to play back the recording, to do the recording again, to discard the recording, or to accept the recording. If accepted, the recording is immediately encrypted on the phone for awaiting subsequent transfer to server. Because Beiwe uses asymmetric encryption, once the recording has been accepted, whether it was played back or not, it is no longer available for playback to the subject.

There is increasing evidence of the clinical value of voice data in schizophrenia in predicting conversation from prodromal state [30], and there is also much literature on the value of correlating such data with changes in symptoms [31,32]. The ability to have subjects record their audio using their smartphones, at a time and place of their choosing, appears to have significant potential to enrich the clinical literature on voice data.

#### Phone and Screen Status

Beiwe records the power state of the phone (screen on/off, power connected/disconnected, and shutdown/restart/boot) and also records all screen touch events, not only those taking place when the subject uses the Beiwe app itself. The latter can be useful for making inferences about subject behavior. For example, data on screen touches can be used to monitor survey response times and to learn whether the subject uses the phone at night to infer coarse sleep duration and quality metrics. See Figure 9 for sample screenshots from the platform.

The clinical significance of such data is broad and one interesting use case is related to cognitive functioning. Knowing how long it takes for a subject to take a survey, something that can be easily quantified from screen touch events, provides novel clinical data about the subject’s cognitive state, and hence about the survey’s validity, that is unavailable from paper-and-pencil scales. Speed of responses to smartphone surveys may be correlated with attention and a useful marker for cognition in many illnesses including schizophrenia or ADHD.

**Figure 8:**
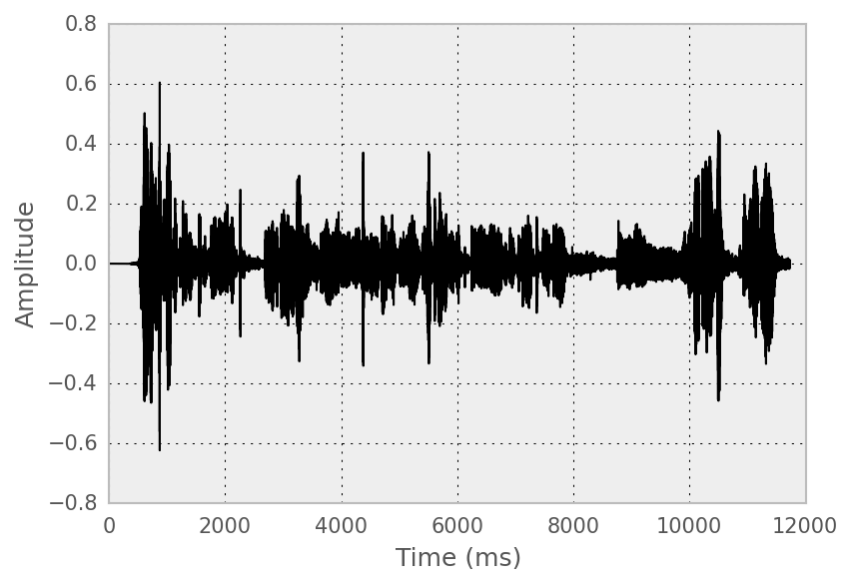
Voice samples are captured in MP4 file format or as raw uncompressed audio data depending on the intended use case.

**Figure 9:**
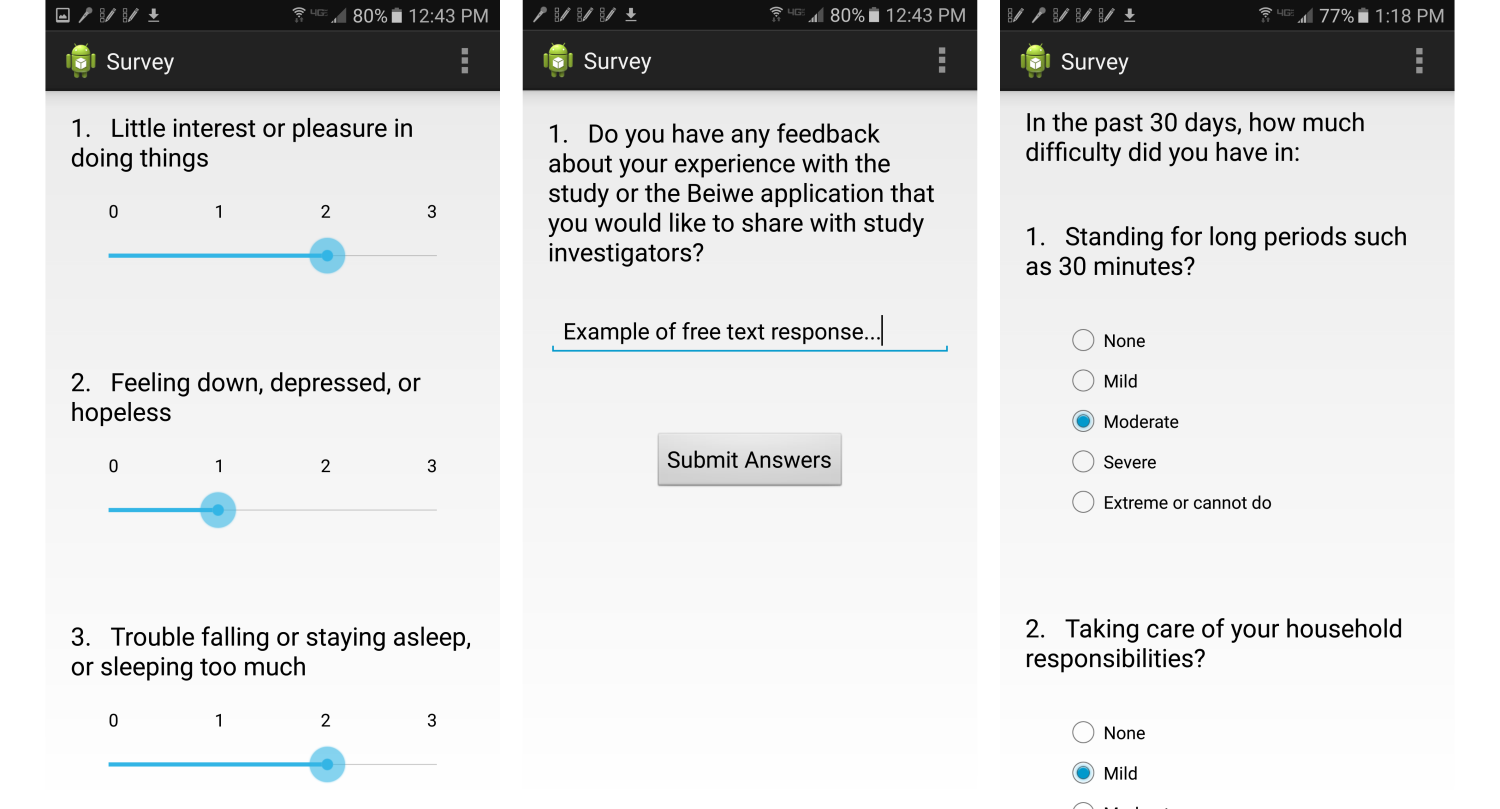
Sample screenshots of customizable surveys that Beiwe is programmed to present to subjects.

## Discussion

### From Data to Biomedical Insights

While we have focused here on the Beiwe smartphone app, it is important to stress that the app is just one of the many components of the Beiwe platform. From our perspective, the key component of the platform is its modeling and data analysis component, which we discuss here only briefly. The development of new biostatistical tools for making sense of smartphone data in the context of digital phenotyping is one of our two main research areas, and we will be documenting our progress in this area in much more detail in journals that focus on the methodology of statistical learning. As we develop and refine these methods, we plan to make the code that implements them open source and accessible to investigators as part of the Beiwe platform, with the ultimate goal of seamlessly integrating data collection and data analysis on a single platform. Transparency in both data collection and data analysis are critical for scientific progress, and Beiwe has been designed specifically for these goals, which differentiates the platform from numerous commercial apps.

Equally important, the Beiwe platform will facilitate re-analyses of existing data and reproducibility of clinical studies. While reproducibility is fundamental to research, it remains a challenge with many existing app platforms. Using Beiwe, it is possible to create validation studies that use the exact same surveys, user prompts, and sensor settings as the original study. This is possible because the platform stores not only the collected raw data, but also the configuration file that specifies all of the app settings. Once the data analysis platform is released more broadly, it will be possible to analyze the data using the same analytical tools that were used in the original. As the analytical tools evolve, we will maintain a complete version history of the software that implements in a Web-based Git repository hosting service, which enables an investigator to match the data with the version of the data analysis modules used in the original study.

Google Flu Trends offers a cautionary tale about lack of reproducibility from a field of research that uses search engine queries to learn about the prevalence of influenza. Although the original study [33] appeared to offer strong support for the use of this approach as a public health surveillance tool, the paper was found to suffer from a lack of reproducibility. This was in part because Google updated its proprietary search algorithm numerous times, up to 86 times in a 2-month period, making even monthly comparisons across studies impossible [34].

The term big data is often used to refer to data that are high velocity (generated continuously), high variety (multiple types of data), and high volume (large quantities of data). Given that the Beiwe app collects data continuously, its data types vary from surveys to audio data, and the net result is a million observations of longitudinal multivariate data being collected per subject per day, it fits squarely within the criteria for big data. We anticipate that the most productive way of analyzing such data might consist of a mixture of more traditional models for longitudinal multivariate data with dimensionality reduction of predictors achieved using machine learning techniques. While at present there is very limited research on big data methods specifically for psychiatric data, there are many promising leads and existing methods and tools that can be applied to such data today [35].

### Outline for Initial Study in Patients With Schizophrenia Spectrum Illness

To better explore the capabilities of the Beiwe platform, assess the app, and produce novel data to fuel the modeling and analytical components of Beiwe, we are in the process of starting a clinical study in patients with schizophrenia. This study has been approved by the Institutional Review Board at Beth Israel Deaconess Medical Center, and it underscores the how the Beiwe platform can be used in clinical research. Schizophrenia is a chronic mental illness characterized by periods of exacerbation of core features including delusions, hallucinations, and disorganized speech and thoughts [36]. The disease has a global impact, afflicting 1.5% of the world’s population [37] and remains one of the most severe illnesses in terms of personal disability [38], suffering [39], economic impact [40], and caregiver burden [41].

Although antipsychotic medications remain the first line treatment for schizophrenia, research suggests that up to 40% of patients discharged from the hospital on an antipsychotic medication may still relapse within 1 year [42]. Common causes of relapse include non-adherence to medications, substance abuse, stress, and disengagement with treatment. The negative consequences of relapse in schizophrenia include decreased quality of life, with relationships and employment often in jeopardy [43], as well as likely neurotoxic effects [44], cognitive decline [45], and often a return to an overall lower level of baseline functioning than prior to relapse [46]. However, there is evidence that early recognition of warning signs of symptom exacerbation and early treatment may mitigate or even prevent relapse [47]. However, because the early warning signs of relapse in schizophrenia may be abrupt [48] and often occur outside of the health care environment, they are often noticed too late when the patient is already psychotic or requires hospitalization [49].

Smartphones and mobile apps offer a novel means of identifying the early signs of symptom exacerbation in schizophrenia outside of the clinic in real life and in real time. Prior research has identified the potential of paper-and-pencil-based EMA in schizophrenia, with an early study noting that half of patients using EMA techniques were able to identify noticeable symptoms of relapse 1 week prior to actual relapse [50]. However, the cumbersome nature of paper-and-pencil EMA, as well as concern for inaccuracy through backfilling [51], has limited its application. Smartphones offer a new means to collect EMA data more easily, and recent research has demonstrated the feasibility of smartphone-based EMA in patients with schizophrenia. Patient feasibility using smartphone apps to self-report symptoms of psychosis has been demonstrated in several studies [19,52,53]. A recent study of 33 patients with schizophrenia noted that patients found the experience of using a smartphone app to provide on-demand psychoeducation in response to self-reported data to be very positive [18]. A survey study of a state mental health clinic serving a majority of patients with psychotic disorders noted that nearly 65% of patients under age 30 may own a smartphone and that overall ownership for all age groups is 33% [3]. Thus, research suggests that patients with schizophrenia are able to use smartphones to monitor their mental health, and many may own smartphones capable of running symptom-monitoring apps.

At present, there is a considerable lack of data regarding the clinical utility of smartphone-based passively collected data streams, like the ones that Beiwe can capture, and their correlations with traditional clinical metrics. Thus, the main objective of our study is to investigate any correlations between active data (here, surveys and audio data), passive data (here, GPS, accelerometer, call and texts logs, and screen event data), and traditional metrics collected in-clinic, with a focus on relapse as well as positive and negative symptoms. There is also a lack of long-term adherence data, with most studies ending after 6 weeks, and consequently little known on how patients learn to use or accept new apps in studies with longer follow-up times [54]. Our secondary objective is thus to study adherence and patterns of app adoption and use over a 3-month period.

In this observational pilot study, we will enroll 20 patients with schizophrenia with inclusion criteria being that they are in current treatment for schizophrenia at the study hospital and own specifically, a smartphone. Age, sex, gender, medication status, or comorbidities will not be exclusion factors. Eligible patients will first be given an in-person assessment consisting of the following validated psychiatric scales: Mini-International Neuropsychiatric Interview (MINI) [55], Patient Health Questionnaire 8 (PHQ-8) [56], General Anxiety Disorder Questionnaire 7 (GAD-7) [57], Pittsburgh Sleep Quality Index (PSQI) [58], and the Warning Signals Scale (WSS), which assess for risk of relapse in schizophrenia [59]. After completing these questionnaires with study staff, patients will be educated on how to download, launch, and use the Beiwe app. Then they will be asked to use it for the next 30 days. The app will be programmed to generate the following surveys: daily WSS and biweekly PHQ-8, PSQI, and GAD-7. The app will also prompt subjects for a daily audio sample. Passive data collection will include GPS, accelerometer, voice call logs, text message logs, and screen event data.

**Figure 10:**
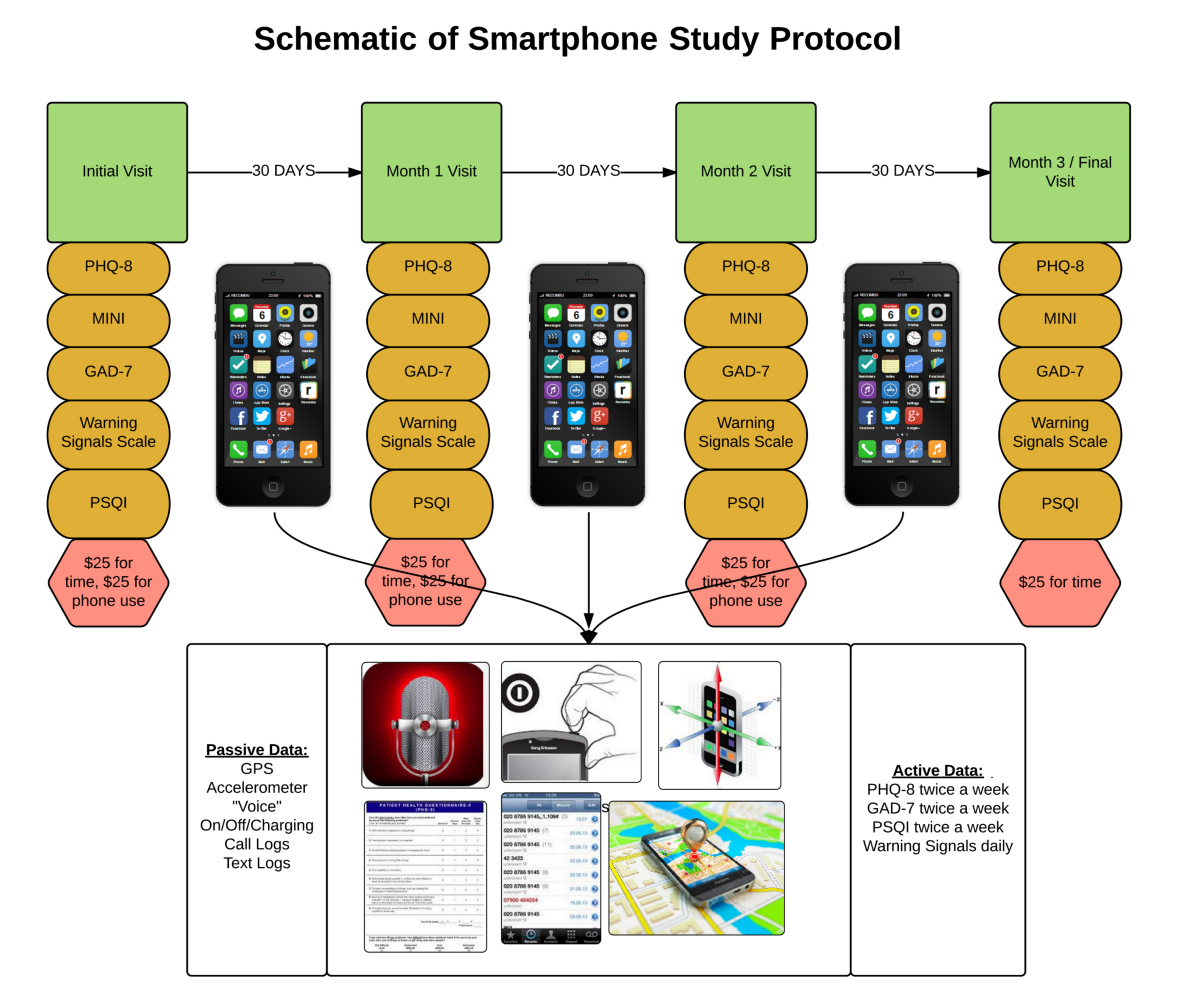
A schematic of the proposed pilot study for patients with schizophrenia using the Beiwe platform.

After 30 days, subjects will be invited back to the clinic for another round of in-person assessment scales identical to those presented at the beginning of the study. Subjects will then be asked to continue using the app for another 30 days and to return at the end of Month 2 for another in-person assessment. Finally, subjects will be asked to continue the use of the app for a yet another 30 days and to return for a final in-person assessment at the end of Month 3. As this is a pilot study, subjects who drop out or leave the study for any reason will not be replaced. See Figure 10 for a schematic of the study design.

We stress that neither Bluetooth nor Wi-Fi data will be collected in this study; although the Beiwe app has the ability to collect these data, these data streams are not central to the clinical question at hand and they do not have an early clinical evidence base yet, unlike the other passive data streams that are included. The customizability of the platform ensures that unnecessary data are not collected and studies can be conducted in as minimally invasive a manner as possible. Just as a study making use of blood samples would collect only the number of samples necessary to run the relevant lab tests, here we also plan to collect only the digital data necessary for the purposes of our study.

Having subjects use their own phones in the study offers several advantages. First, it may likely reduce bias and the Hawthorne effect, as studies of psychiatric patients have reported patients being more comfortable with their own devices [19,60]. Second, using subjects’ own smartphones reduces study costs. In this pilot study, subjects will be paid only for their time for completing in-person clinical assessments and will be reimbursed a flat rate for using their own smartphone in the course of the study. Note that the patients do not have to be compensated for cell phone data use as all data uploads happen through Wi-Fi. Third, for mobile mental health interventions to be scalable and impactful, especially in the long term, they will likely have to rely on patients’ own smartphones, and thus it makes sense to have patients use their own phones also in our pilot. Of note, in our internal testing of the Beiwe platform customized for this study, we did not notice any considerable drain on battery or change in the phone’s performance or speed based on the daily feedback of numerous healthy volunteers. While in principle it is possible that a subject never connects to a Wi-Fi network and thus data collection would be limited by the phone’s internal storage space, in practice this has never happened in either test use or in our other ongoing studies that use the same platform.

This methodology of collecting patient self-reported symptoms in the form of smartphone surveys complemented with behavioral data from smartphone sensors, which includes physiological data related to sleep patterns and voice features, matches the NIMH’s Research Domain Criteria (RDoc) model, which encourages the simultaneous examination of multiple levels of the system. The results from this study could provide novel insights to the utility of big data in psychiatry and demonstrate how digital phenotyping more broadly may contribute to our understanding of schizophrenia.

### Conclusion

With Beiwe, we introduce a new platform that will enable digital phenotyping in a scalable, customizable, transparent, and reproducible manner. The platform consists of a Web-based study portal used to manage studies, a smartphone app customized to the needs of any given study, backend database for storing study data and metadata, and a suite of software to be used to model and analyze the data collected using the platform. At the time of writing, the first version of the data analysis and modeling component is being developed, with all other components being fully functional. While there are other smartphone apps that are used to collect both active and passive data from patient cohorts, the Beiwe platform features the collection of high-quality raw data from smartphones and couples this with appropriate statistical learning tools that can be readily applied to the collected data. Because both data collection and data analysis are carried out using tools that will be released to the scientific community, we expect this open design paradigm to foster a more productive and more sustainable approach to digital phenotyping than reliance on proprietary black box tools.

While at this stage we are studying the clinical utility and validity of Beiwe in patients with schizophrenia, and in this paper have focused on the app component of the platform, we are in the process of evolving and expanding out the data analysis and methods component to match the nature and demands of complex data generated by the platform. We are currently using Beiwe in several other clinical studies in the Boston metropolitan area and exploring new avenues, such as incorporating wearable sensor data and DNA sequencing data, to realize the full potential of digital phenotyping.

We hope that this brief introduction to the Beiwe platform will be a step to directing the present discussion about smartphone and mobile apps as data collection tools toward a discourse centered on the new kinds of science that they may enable and the new kinds of data analytical approaches that will be needed to redeem their full potential.

## Abbreviations

EMA: ecological momentary assessment
ESM: experience sampling method
GAD-7: General Anxiety Disorder Questionnaire 7
NIMH: National Institute of Mental Health
PHQ-8: Patient Health Questionnaire 8
PSQI: Pittsburgh Sleep Quality Index
RSA: Rivest-Shamir-Adleman
WSS: Warning Signals Scale
